# Glycerol Supplementation Enhances *L. reuteri*’s Protective Effect against *S.* Typhimurium Colonization in a 3-D Model of Colonic Epithelium

**DOI:** 10.1371/journal.pone.0037116

**Published:** 2012-05-31

**Authors:** Rosemarie De Weirdt, Aurélie Crabbé, Stefan Roos, Sabine Vollenweider, Christophe Lacroix, Jan Peter van Pijkeren, Robert A. Britton, Shameema Sarker, Tom Van de Wiele, Cheryl A. Nickerson

**Affiliations:** 1 Laboratory of Microbial Ecology and Technology (LabMET), Ghent University, Ghent, Belgium; 2 Center for Infectious Diseases and Vaccinology - The Biodesign Institute, Arizona State University, Tempe, Arizona, United States of America; 3 Department of Microbiology, Uppsala BioCenter, Swedish University of Agricultural Sciences, Uppsala, Sweden; 4 Institute of Food, Nutrition and Health, ETH Zürich, Zürich, Switzerland; 5 Department of Microbiology & Molecular Genetics, Michigan State University, East Lansing, Michigan, United States of America; Indian Institute of Science, India

## Abstract

The probiotic effects of *Lactobacillus reuteri* have been speculated to partly depend on its capacity to produce the antimicrobial substance reuterin during the reduction of glycerol in the gut. In this study, the potential of this process to protect human intestinal epithelial cells against infection with *Salmonella enterica* serovar Typhimurium was investigated. We used a three-dimensional (3-D) organotypic model of human colonic epithelium that was previously validated and applied to study interactions between *S.* Typhimurium and the intestinal epithelium that lead to enteric salmonellosis. Using this model system, we show that *L. reuteri* protects the intestinal cells against the early stages of *Salmonella* infection and that this effect is significantly increased when *L. reuteri* is stimulated to produce reuterin from glycerol. More specifically, the reuterin-containing ferment of *L. reuteri* caused a reduction in *Salmonella* adherence and invasion (1 log unit), and intracellular survival (2 log units). In contrast, the *L. reuteri* ferment without reuterin stimulated growth of the intracellular *Salmonella* population with 1 log unit. The short-term exposure to reuterin or the reuterin-containing ferment had no observed negative impact on intestinal epithelial cell health. However, long-term exposure (24 h) induced a complete loss of cell-cell contact within the epithelial aggregates and compromised cell viability. Collectively, these results shed light on a potential role for reuterin in inhibiting *Salmonella*-induced intestinal infections and may support the combined application of glycerol and *L. reuteri*. While future *in vitro* and *in vivo* studies of reuterin on intestinal health should fine-tune our understanding of the mechanistic effects, in particular in the presence of a complex gut microbiota, this the first report of a reuterin effect on the enteric infection process in any mammalian cell type.

## Introduction

Intestinal infection by non-typhoidal *Salmonella* strains (NTS) leads to gastroenteritis and diarrheal disease, and is a major source of foodborne illness worldwide [1]. *Salmonella enterica* serovar Typhimurium (*S.* Typhimurium) causes a self-limiting gastroenteritis and diarrheal disease in healthy individuals, but can cause life threatening systemic illness in immuno-compromised individuals. Usually, treatment relies on water and electrolyte supplementation, but when the associated dehydration becomes severe or when *Salmonella* reaches the bloodstream, antimicrobial treatment becomes essential. However, since the 1990s multi-drug resistant strains have emerged and are compounding the problems associated with severe enteric salmonellosis [2], [3]. Therefore, the development of novel therapeutic treatments and vaccines is ongoing. An alternative approach is to prevent or reduce *Salmonella* infection, using benign microorganisms that are naturally occurring in the gastrointestinal tract. The gut symbiont *Lactobacillus reuteri* has previously been shown to protect against gastrointestinal infections and even to reduce diarrhea in children [4], [5]. However, it remains unclear how these effects are mediated. In this study, we explored the possibility for *L. reuteri* to decrease *S.* Typhimurium infection of the intestinal epithelium by producing and excreting the antimicrobial metabolite reuterin during the infection process.

Reuterin or the hydroxypropionaldehyde (HPA)-system is an antimicrobial mixture of 3-HPA mono-, di- and polymers, and HPA-hydrate. It is produced as an intermediate during the reduction of glycerol to 1,3-propanediol (1,3-PDO) [6]. Several gut bacterial species are described to produce reuterin in a specialized bacterial compartment or metabolosome [7]. Among these species, *L. reuteri* is the one reported to most efficiently produce reuterin and excrete relatively high amounts [8]–[10]. Only recently, two studies demonstrated that reuterin’s mode of action involves the modification of thiol groups in proteins and small molecules, which results in oxidative stress and ultimately leads to bacterial cell death [11], [12]. Moreover, the latter studies indicated that the aldehyde group in 3-HPA is the bioactive agent in reuterin.

Several studies have investigated the antimicrobial activity of reuterin by determining its minimal inhibitory and bactericidal concentration (MIC and MBC) on pathogens and commensal gut bacteria both in the absence and presence of *L. reuteri* [13]–[17]. Recent reports indicated that reuterin production also occurs in mixed microbial communities found in human feces [18], [19], whereas Morita *et al.* [20] found *in vivo* reuterin production in the caecum of gnotobiotic mice inoculated with *L. reuteri*. Furthermore, there is increasing evidence that reuterin production is crucial for the ecological behavior and probiotic properties of *L. reuteri* isolates from the human gastrointestinal tract [21], [22]. While mechanistic *in vitro* studies demonstrated that *L. reuteri* may protect intestinal cells against enteric infection by regulating immune responses [23]–[27] or by competition for host binding sites [28], the effect of reuterin production on modulating host-pathogen interactions with eukaryotic cells has not yet been investigated. Hence, in this study we assessed the capacity of reuterin to target the early stages of *S.* Typhimurium interaction with human colonic epithelium that lead to enteric salmonellosis, specifically adherence and invasion, intracellular survival and intracellular growth (i.e. colonization).

For these studies, we utilized a previously characterized rotating wall vessel (RWV)-derived 3-D HT-29 organotypic model of human intestinal epithelium. The application of a 3-D HT-29 model for this investigation is logical, since the former better approximates the parental tissue and has been shown to be more predictive of key *in vivo* responses to infection by *S.* Typhimurium as compared to monolayers [29], [30]. This is evidenced by 3-D HT-29 cells exhibiting a) distinct apical and basolateral polarity, b) enhanced expression and organization of tight junctions, extracellular matrix, and brush border proteins, c) highly localized mucus production, and d) differentiation into multiple epithelial cell types relevant to those found in the intestine, including enterocytes, goblet cells, Paneth cells and M-like cells [29]–[31]. These *in vivo*-like expression levels and distribution patterns of key biological surface markers in 3-D cells are critical for differentiated form and function. These traits of the 3-D models could contribute to their ability to support pathogen infection in a manner more akin to that in the infected host as compared to monolayers [31]. For example, the enhanced formation, integrity, and physiological distribution of tight junctions in 3-D HT-29 epithelial cells could serve as a protective barrier against pathogen invasion, thereby reducing infection and maintaining structural integrity. In addition, differences in mucus localization could alter bacterial adherence and invasion into the underlying epithelium. Moreover, the differentiation status of the intestinal epithelium drives important differences in cytokine production in polarized cells following bacterial infection as compared to their non-polarized counterparts, which is also an important determinant of infection outcome [32], [33]. Likewise, the use of human surrogate infection models that possess multiple epithelial cell types normally found in the intestine is essential to better predict *in vivo*-like responses to infection with enteric pathogens. When applied to study the early stages of human enteric salmonellosis, the highly differentiated 3-D HT-29 intestinal model responded in ways that were similar to an *in vivo* infection, including differences in tissue morphology, adherence, invasion, apoptosis and cytokine expression [29], [31]. To our knowledge, 3-D HT-29 cells were the first *in vitro* model of human intestinal epithelium to suggest that the *Salmonella* Pathogenicity Island 1 Type Three Secretion System (SPI-1 T3SS) may not be the main determinant for invasion of *Salmonella* into *in vitro* models of human intestinal epithelium [29]. Subsequent work demonstrated that all characterized *S.* Typhimurium T3SSs (SPI-1, SPI-2, and the flagellar pore) are dispensable for *Salmonella* invasion into highly differentiated 3-D HT-29 cells, but are required for intracellular bacterial growth, paralleling *in vivo* infection observations and demonstrating the utility of these models in predicting *in vivo-*like pathogenic mechanisms and for studying host-microbe interactions [30]. Using this organotypic model, we show that glycerol conversion to reuterin by *L. reuteri* reduces *S.* Typhimurium colonization of human intestinal epithelium and might be a useful therapeutic approach to consider for treatment and prevention of enteric salmonellosis.

## Methods

### Bacterial Strains, Media and Growth Conditions

The strains used in this study are provided in Table 1. *L. reuteri* ATCC PTA 6475 belongs to lineage II of the *L. reuteri* species [34]. The genome of lineage II *L. reuteri* contains the *pdu-cbi-cob-hem* gene cluster, which encodes reuterin production [22]. A knock-out mutant of *pduC* was generated using RecT-mediated oligonucleotide recombineering, as described by van Pijkeren and Britton [35]. *L. reuteri* expressing RecT (strain RPRB0000) was used to target the *pduC* gene (Genbank locus ZP_08162814; glycerol dehydratase, large subunit) to yield strain RPRB1321, further indicated as ATCC PTA 6475 Δ*pduC*. Mutations were verified by PCR, and the integrity was confirmed by sequence analysis. Oligonucleotides for recombineering and screening purposes are available upon request.

**Table 1:** Overview of the bacterial strains used in this study.

Bacterial strains	Description	Source
*Salmonella enterica* serovar Typhimurium χ3339	Animal-passaged isolate of the virulent SL1344 wild-type strain	[65]
*Escherichia coli* HB101	Non-invasive extracellular	[66]
*L. reuteri* ATCC PTA 6475	Isolate from Finnish mother’s milk	BioGaia AB(Raleigh, USA)
RPRB0000	*L. reuteri* expressing RecT	[35]
RPRB1321– ATCC PTA 6475 Δ*pduC*	*L. reuteri::oJP859* (*pduC* ¶: W24X, P25I, E26Q)*pduC* knockout, unable to produce glycerol dehydratase	This study

¶ : *pduC*: HMPREF0536_1321. Resultant amino acid changes after incorporation of the oligonucleotide are listed after each gene, and X represents a stop codon.

**Figure 1:**
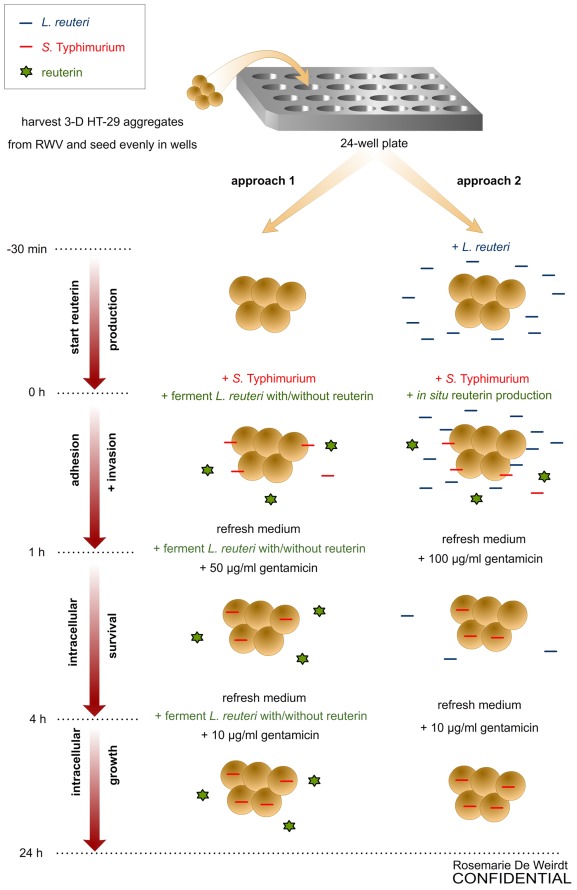
Experimental procedure for infection of 3-D HT-29 aggregates with *S.* Typhimurium χ3339 (red line). Approach 1 infection experiments were done in the presence of supernatants of the *L. reuteri* ferment with or without reuterin (green star), while approach 2 infections were done in the presence of an established *L. reuteri* population (blue line) producing reuterin *in situ*.

*S.* Typhimurium and *E. coli* were grown on Lennox (L) agar (Fisher, New Jersey, USA) and *L. reuteri* was grown on de Man-Rogosa-Sharpe (MRS) agar (Difco, Maryland, USA) for 24 h at 37°C. Then, a colony of *S.* Typhimurium and *E. coli* was picked up and grown overnight (for approximately 15 h) in 5 mL L broth at 37°C with shaking at 180 rpm. Prior to use in experiments, overnight cultures were back-diluted 1∶100 in L broth and grown at 37°C with aeration until reaching mid-log phase of growth (OD595 ≈ 0.6). All infection studies were performed at a multiplicity of infection (MOI) of approximately 1 bacterium per host cell. Similarly, a colony of *L. reuteri* ATCC PTA and ATCC PTA 6475 Δ*pduC* was picked and grown overnight (16–18 h) in 5 mL MRS broth either without or with 20 mM glycerol (99.9%, Sigma, Missouri, USA) at 37°C with shaking at 180 rpm. Ferments of ATCC PTA 6475 were prepared by harvesting (1500 rcf for 10 min) and washing the pellets with 5 mL Dulbecco’s Phosphate Buffered Saline (DPBS) with ions, and incubating them in 5 mL of the mammalian cell culture medium GTSF-2 without or with 100 mM glycerol during 2 h at 37°C with shaking at 180 rpm. The supernatant of these ferments was recovered after centrifugation at 8000 rcf for 10 min and filter-sterilized over a 0.45 µM PVDF filter (Whatman, Maine, USA). Prior to use in approach 1 experiments (Fig. 1), the supernatant obtained by fermentation without glycerol was diluted 10-fold in GTSF-2 medium (SN- 1∶10) and the supernatant of the ferment with glycerol was diluted 10-, 100- and 1000-fold, further indicated as SN+1∶10, SN+1∶100 and SN+1∶1000. For use in approach 2 infection experiments (Fig. 1), ATCC PTA 6475 was grown either in the absence or presence of 20 mM glycerol, while ATCC PTA 6475 Δ*pduC* was grown in the absence of glycerol. Consecutively, the pellets were suspended in 5 mL GTSF-2 without (PTA 6475 -) or with 40 mM glycerol (PTA 6475+ and PTA 6475 Δ*pduC* +). The *L. reuteri* cultures were diluted twice prior to use in infection studies, resulting in a population of (1.8±0.1)×10^9 ^CFU.mL^−1^.

To our knowledge, there are no data available on actual glycerol and reuterin concentrations in the human gut *in vivo*. However, Morita *et al.* [20] were the first to demonstrate that reuterin can be produced *in vivo* in the intestine of mice and that feces of untreated mice contain glycerol concentrations of 7–10 mM, suggesting the presence of glycerol in the intestine. Moreover, glycerol may rapidly be consumed by the colon microbiota [18], [19], which indicates that actual glycerol concentrations in the intestine might be higher than those found in the feces.

### Reuterin Production and Quantification of Glycerol Metabolites

Reuterin was produced by *L. reuteri* ATCC 53608 in a two-step glycerol fermentation process and purified to a stock of 330 mM 3-HPA as described previously [10]. Glycerol, acetic acid, lactic acid and 1,3-PDO were quantified by means of HPLC-UV/RI. Detailed information on sample preparation and analytical parameters can be found in [19]. The concentration of 3-HPA was determined in the fresh samples by means of a colorimetric assay previously described by Vollenweider *et al.* [10] and modified to a 96-well plate format by Spinler *et al.* [16]. In brief, 50 µL of a (10-fold diluted) sample was mixed with 37.5 µL tryptophan solution (0.01 M in 0.05 M HCl, stabilized with a few drops of toluene per 250 mL) and 150 µL 12 M HCl, and incubated during 20 min at 37°C. Then, reuterin was quantified as 3-HPA based on the optical density measured at 550 nm and a standard curve made with acrolein (Fluka, St. Louis, MO, USA).

### Growth Inhibition of *S.* Typhimurium by 3-HPA and Supernatants from the *L. Reuteri* Ferment

To assess the effects of the supernatants from the ATCC PTA 6475 ferment (SN- and SN+) and 3 mM 3-HPA on survival and growth of *S.* Typhimurium, parallel experiments were performed according to the approach 1 infection studies described below and depicted in Fig. 1. The experiments were set-up in 24-well plates (500 µL/well) in the absence of host cells and with a single addition of the supernatants from the ATCC PTA 6475 ferment in GTSF-2. Mid-log phase grown *S.* Typhimurium was added to the wells at a concentration of 2×10^6^ cells.mL^−1^ and incubated statically at 37°C in a 5% CO_2_ environment. A 20-µL sample was taken to plate 10-fold dilution series on L agar plates after 1, 4 and 24 h. The experiment was repeated three times and each time performed in duplicate, resulting in two technical and three biological replicates.

The minimal inhibitory concentrations (MICs) of 3-HPA and the reuterin-containing supernatants of the ATCC PTA 6475 ferment were determined for *S.* Typhimurium χ3339 using a standard serial dilution overnight growth assay downscaled to a 96-well plate. In brief, 2-fold dilution series were made in GTSF-2 starting either from 33 mM 3-HPA (20 µL 3-HPA stock of 330 mM in milliQ +180 µL GTSF-2) or undiluted supernatant of the ATCC PTA 6475 ferment. The final volume in each well was 100 µL. Then, a mid-log phase grown *S.* Typhimurium culture was added to the wells in a maximum volume of 3.8 µL per well and a final concentration of 2×10^6^ bacterial cells.mL^−1^. The MIC was determined to be an intermediate concentration lying between that of the wells with and without visible growth (turbidity) after 24 h of static incubation at 37°C. The assay was repeated two times from independent *S.* Typhimurium cultures, each including 3 replicates.

### 3-D Model of Colonic Epithelium

Three-dimensional intestinal models were derived from the human colonic epithelial cell line HT-29 (American Type Culture Collection ATCC # HTB-38), using the RWV bioreactor from Synthecon as previously described [29], [30]. For all studies, HT-29 cells were cultured in GTSF-2 medium (Hyclone) supplemented with heat-inactivated fetal bovine serum (Invitrogen, California, USA), ITS (insulin-transferrin-sodium selenite; Sigma, Missouri, USA), sodium bicarbonate, penicillin/streptomycin (Sigma, Missouri, USA) and fungizone (Invitrogen, California, USA) as described by Bentrup *et al.* [29]. Fresh medium was replenished after 5 days and then every 24 h until harvest of the cultures after 15–18 days.

### Adherence and Invasion, Intracellular Survival and Intracellular Growth Assays

A gentamicin exclusion assay was performed to assess *S. *Typhimurium adhesion and invasion (1 h), intracellular survival (4 h) and intracellular growth (24 h). The experimental flow was modified from previous publications [29], [30], [36] and is depicted in Fig. 1. Two days prior to the infection experiments, 3-D HT-29 cells were grown in antibiotic free GTSF-2, and streak incubations of the bacterial cultures were started on agar and further prepared as described above. On the day of infection (day 15–18), the 3-D aggregates were removed from the RWV bioreactor and seeded evenly into 48-well plates with 2×10^6^ cells.mL^−1^ and 250 µL GTSF-2 per well. The number of cells associated with 3-D aggregates was determined by dissociation into individual cells with 0.25% trypsin-EDTA. The cell number and viability were determined by 0.4% trypan blue (1∶1) (Sigma, Missouri, USA) dye exclusion and counting in a hemocytometer. *S.* Typhimurium or non-invasive *E. coli* were added to the 3-D HT-29 cells at an MOI of approximately 1-to-1 (bacteria-to-host cell). In approach 1 infection experiments, 3-D aggregates were exposed to *S. *Typhimurium or *E. coli* together with (a) 3-HPA (3 mM) or (b) dilutions of the supernatants from the *L. reuteri* ferment as described above. Infections according to approach 2 were done in the presence of an established (reuterin-producing) *L. reuteri* population. Non-exposed controls (with and without bacteria) were included. Both approach 1 and 2 cultures were incubated for 1 h in a 5% CO_2_ environment at 37°C, yielding adhered and invaded *S.* Typhimurium. Medium was subsequently changed to GTSF-2 containing gentamicin to eliminate extracellular bacteria, and cells were incubated for an additional 3 h. Then, the intracellular bacteria were determined (survival) and again fresh medium with gentamicin was added for a final incubation until 24 h after the start of infection to determine intracellular growth. At each time point, adherent and/or intracellular bacteria were quantified after lysing the host cells with 0.1% sodium deoxycholate (approach 1) or 1% triton (approach 2) and plating serial dilutions of the lysates on L agar plates overnight. Additionally, host cell viability was assessed following trypsinization of the 3-D aggregates, staining with 0.4% trypan blue (1∶1) (Sigma, Missouri, USA) and counting in a hemocytometer. All infection experiments were performed at least twice, in duplicates, resulting in a minimum of two technical and two biological replicates.

### Statistical Analysis

Significant differences between treatments were detected with SPSS Statistics 19. Normality was tested with the Kolmogorov-Smirnoff test. Normal distributed data were analyzed using one-way ANOVA, testing for equality of the variances with the Modified Levene test and determining the p-value according to Bonferroni or Dunnett T3. Significant differences within non-normal distributions were detected using the Mann-Whitney U test. The significance level was set at 0.05.

## Results

### Characterization of the Supernatants from the *L. Reuteri* Ferment

A fully-grown culture of *L. reuteri* ATCC PTA 6475 was incubated in GTSF-2 without (SN-) and with 100 mM glycerol (SN+) for 2 h. The supernatant of these ferments was characterized for its glycerol, 3-HPA, 1,3-PDO, acetic acid and lactic acid content (Table 2). Prior to application in growth and infection experiments the SN- supernatant was diluted 10-fold and the SN+ supernatant 10-, 100- or 1000-fold. In the SN+ supernatant, 67±4 mol % of the supplemented glycerol had been converted to 3-HPA (62±7% of converted glycerol) and 1,3-PDO (42±6% of converted glycerol). Acetic acid and lactic acid were formed in both the SN- and the SN+ supernatant, but in different amounts. The lactic acid concentration was significantly higher in the SN- supernatants (p<0.001), while the acetic acid concentration was more than 3-fold higher in the SN+ supernatants (p<0.001). The pH of all dilutions was very similar with an average value of 7.49±0.02.

**Table 2:** Quantification of the glycerol, 3-hydroxypropionaldehyde (3-HPA), 1,3-propanediol (1,3-PDO), acetic acid and lactic acid contents (mM) in the supernatant of the *L. reuteri* ferments without (SN-) or supplemented with 100 mM glycerol (SN+).

mM	*L. reuteri* ferment noGlycerol (SN-)	*L. reuteri* ferment+100 mM glycerol (SN+)
Glycerol	*bdl*	32.8±3.6
3-HPA	*bdl*	24.5±4.0
1,3-PDO	*bdl*	38.7±3.2
acetic acid	4.0±0.6	13.6±0.5
lactic acid	13.3±0.5	9.7±0.4

*bdl:* below detection limit (1.1 mM for glycerol; 0.8 mM for 3-HPA and 1.3 mM 1,3-PDO).

### Growth Effects of Supernatants from the *L. Reuteri* Ferment and Pure 3-HPA on a *S.* Typhimurium Population

**Figure 2:**
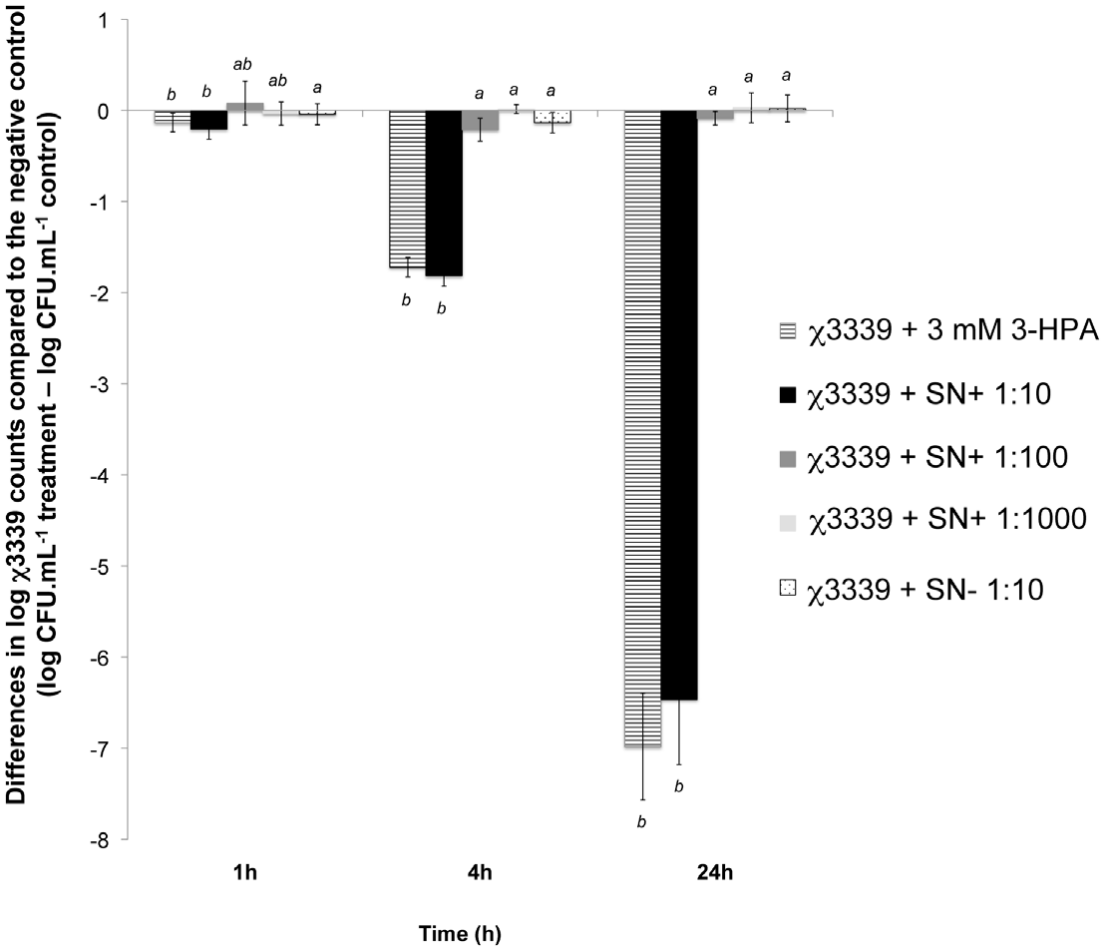
3-HPA and the 10-fold diluted reuterin-containing supernatant significantly decreased the *S.* Typhimurium χ3339 population. Log difference in χ3339 counts (CFU.mL^−1^) of the untreated χ3339 control minus the treatments with 3 mM 3-HPA, the 10-, 100- or 1000-fold diluted supernatants containing reuterin (respectively SN+1∶10 with 2.5 mM 3-HPA, SN+1∶100; 0.25 mM 3-HPA and SN+1∶1000; 0.025 mM 3-HPA) and the 10-fold diluted supernatant without reuterin (SN- 1∶10) after 1, 4 and 24 h of exposure. Significant differences between the treatments are indicated with different letters (a or b; p<0.05).

The growth effects of the supernatant from the *L. reuteri* ferments on the *S.* Typhimurium population were assessed after 1, 4 and 24 h of exposure to dilutions of the ferment, parallel to the approach 1 infection assay. Fig. 2 shows the reduction of the χ3339 population after exposure to the 10-fold diluted reuterin-containing supernatant (SN+1∶10, 2.5±0.4 mM 3-HPA) with 0.2±0.1 (1 h), 1.8±0.1 (4 h) and 6.5±0.7 (24 h) log units, respectively. Addition of 3 mM 3-HPA resulted in a similar reduction of the χ3339 population (0.1±0.1, 1.7±0.1 and 7.0±0.6 for 1, 4 and 24 h, respectively). No significant growth effects were observed for higher dilutions of the same supernatant (SN+1∶100 and SN+1∶1000) or the supernatant without reuterin (SN- 1∶10).

The MIC-value of the supernatants from the reuterin-containing ferment of *L. reuteri* corresponded to a 3-HPA concentration between 0.9 and 1.4 mM, as calculated from the MIC-values obtained for two biological replicates. The MIC-value of pure 3-HPA was found to lie between 1.0 and 2.1 mM.

### Infection of 3-D Intestinal Cells with *S.* Typhimurium in the Presence of Supernatants from the *L. Reuteri* Ferment or Pure 3-HPA (Approach 1)

After assessment of the growth effects of the *L. reuteri* supernatant and pure 3-HPA on *S.* Typhimurium χ3339, their capacity to generate similar effects in the presence of eukaryotic cells and to protect the intestinal epithelium against *S.* Typhimurium infection was tested. This approach allowed us to more closely assess the effect of reuterin on the *in vivo* infection process of *S. *Typhimurium. Therefore, 3-D HT-29 cells infected with *S. *Typhimurium were exposed to *L. reuteri* supernatant and pure 3-HPA under the same conditions of incubation (medium, temperature, CFU.mL^−1^, 3-HPA concentration) as for the experiments with *S.* Typhimurium in the absence of host cells.

**Figure 3:**
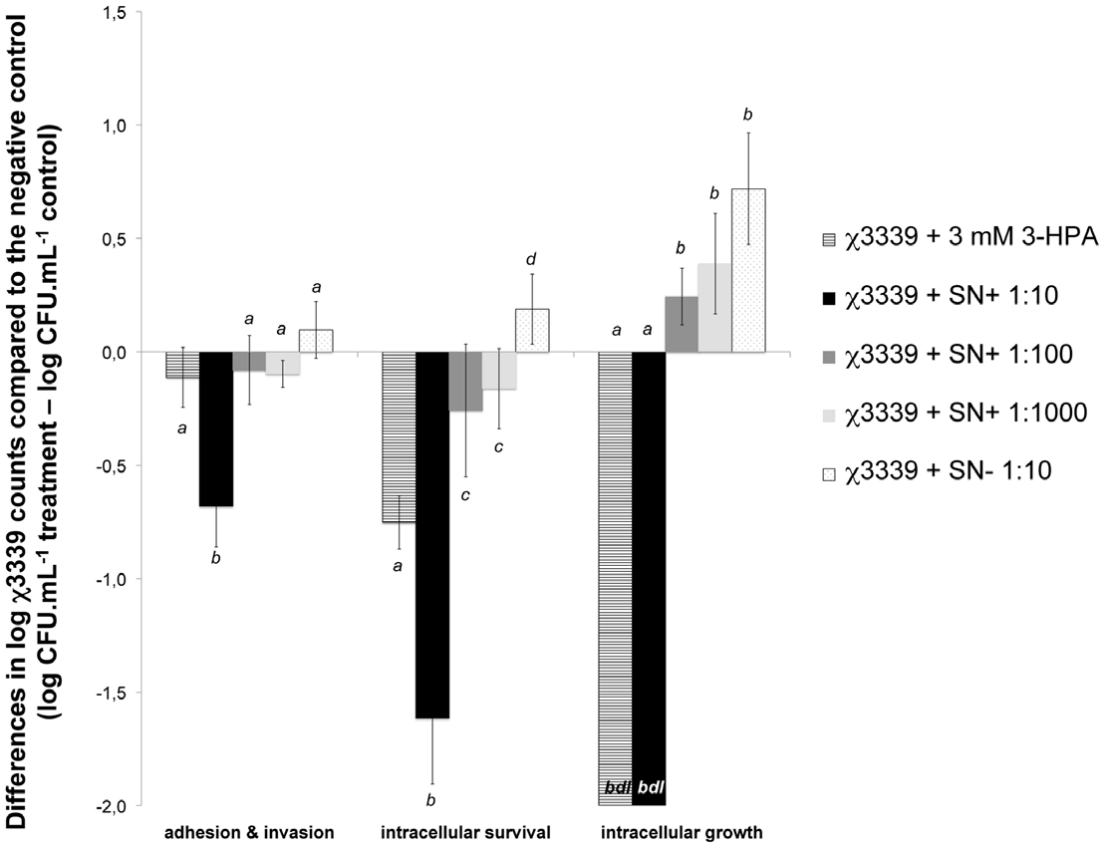
The 10-fold diluted reuterin-containing supernatant decreased *S.* Typhimurium χ3339 colonization in 3-D HT-29 aggregates, while the supernatant without reuterin stimulated χ3339 intracellular survival and growth. Log difference in χ3339 counts (CFU.mL^−1^) of the untreated χ3339 control minus the treatments with 3 mM 3-HPA, the 10-, 100- and 1000-fold diluted supernatants containing reuterin (respectively SN+1∶10 with 2.5 mM 3-HPA, SN+1∶100; 0.25 mM 3-HPA and SN+1∶1000; 0.025 mM 3-HPA) and the 10-fold diluted supernatant without reuterin (SN- 1∶10) after 1 h (adhesion & invasion), 4 h (intracellular survival) and 24 h (intracellular growth) of exposure. The non-invasive *E. coli* HB101 control strain did not show countable colonies at the lowest dilution (detection limit = 10^2^ CFU.mL^−1^). Significant differences between the treatments are indicated with different letters (a, b, c or d; p<0.05). *bdl* = below detection limit.

The 10-fold diluted reuterin-containing supernatant (2.5±0.4 mM 3-HPA) from the *L. reuteri* ferment was able to significantly reduce χ3339 adhesion and invasion, and intracellular survival with 0.7±0.2 and 1.6±0.3 log units, respectively, when compared to the untreated *S.* Typhimurium control (Fig. 3). Moreover, after 24 h no χ3339 survival was detected (detection limit = 10^2**^CFU.mL^−1^). Higher dilutions of the reuterin-containing supernatants did not result in any statistically significant differences compared to the control (Fig. 3). Addition of 3 mM 3-HPA resulted in a 0.8±0.1 log unit reduction of the χ3339 intracellular survival (Fig. 3). However, it did not affect χ3339 adhesion and invasion. Interestingly, the 10-fold diluted *L. reuteri* supernatant without reuterin was found to stimulate χ3339 adhesion and invasion, and intracellular survival, with a 0.7±0.2 log unit increase in intracellular growth after 24 h (Fig. 3).

**Figure 4:**
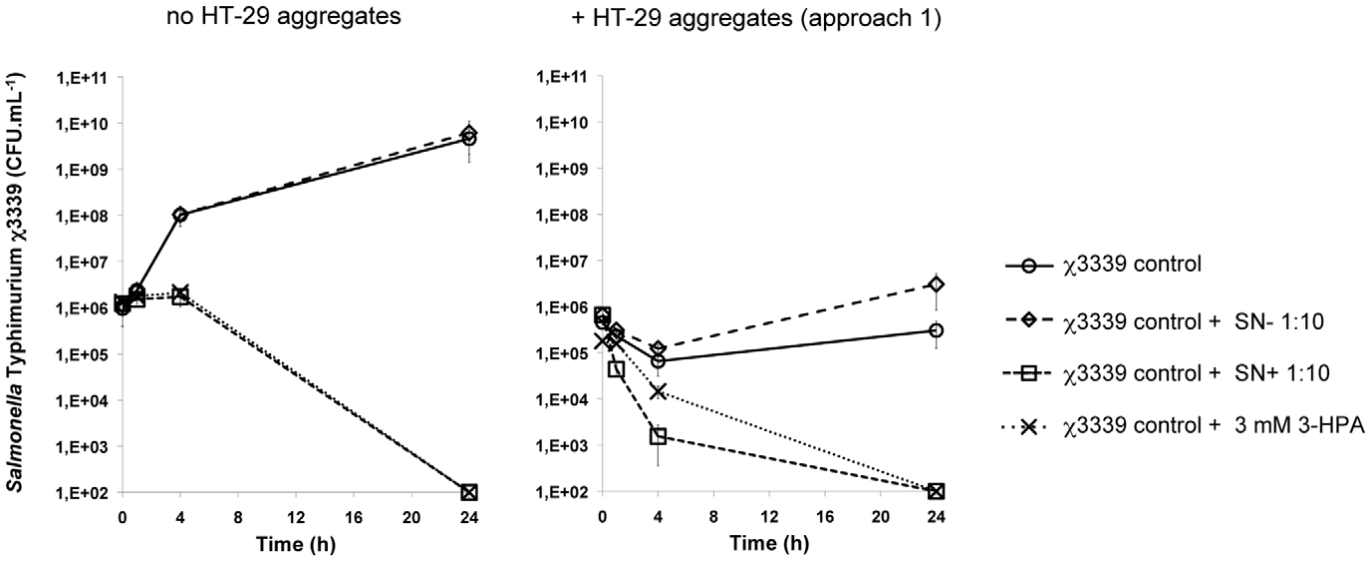
The *L. reuteri* supernatants affected the *S.* Typhimurium χ3339 population stronger in the presence of the 3-D HT-29 aggregates. Log counts (CFU.mL^−1^) of the χ3339 population (initial inoculum 2×10^6^ cells.mL^−1^) exposed to the 10-fold diluted supernatant without (SN- 1∶10) and with reuterin (SN+1∶10, 2.5 mM 3-HPA) both in the absence (left) and presence (right) of 3-D HT-29 aggregates. Detection limit = 10^2^ CFU.mL^−1^.

In Fig. 4, the effects of 3 mM 3-HPA and the supernatants of the *L. reuteri* ferments on χ3339 adhesion and invasion, intracellular survival and growth are compared with those found when no HT-29 aggregates were added (Fig. 2). In the presence of the HT-29 aggregates, both supernatants seemed to affect the χ3339 population stronger. For the reuterin-containing supernatant (SN+1∶10; 2.5 mM 3-HPA) and 3 mM pure 3-HPA, a bacteriostatic effect was observed up to 4 h of exposure in the absence of the HT-29 aggregates, while in the presence of these aggregates, the reuterin-containing supernatant – but not pure 3-HPA – immediately affected the χ3339 population viability after 1 h. In addition, the supernatants without reuterin (SN- 1∶10) significantly stimulated χ3339 intracellular growth in the presence of HT-29 aggregates, while no such effect was observed for the SN- 1∶10 supernatant in the absence of the aggregates.

The negative control strain, *Escherichia coli* HB101, did not exhibit invasion into the colon cells, as no bacterial colony forming units were detected after addition of gentamicin to the medium (4 h) (detection limit = 10^2^ CFU.mL^−1^).

### Infection of 3-D Intestinal Cells with *S.* Typhimurium in the Presence of an Established *L. Reuteri* Population (Approach 2)

**Figure 5:**
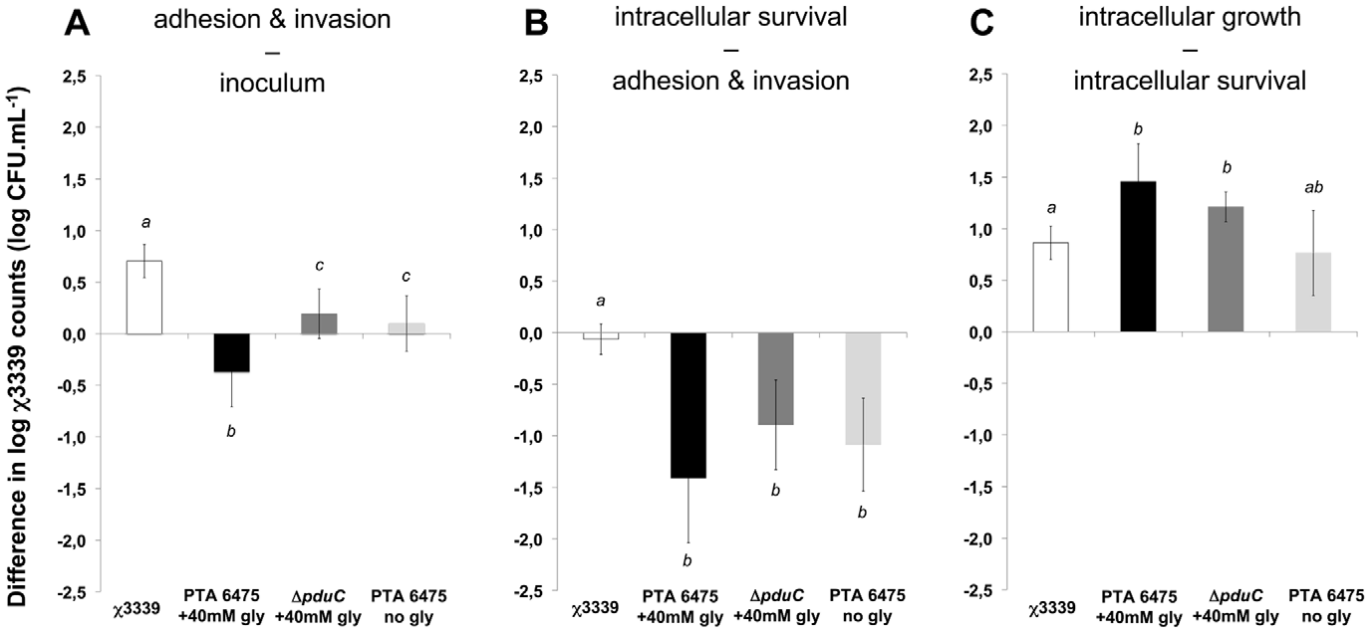
The presence of an established *L. reuteri* population reduced χ3339 adhesion and invasion in 3-D HT-29 aggregates, and this effect was significantly higher when *L. reuteri* was producing reuterin *in situ*. Log differences in χ3339 counts (CFU.mL^−1^) of (A) adhered and invaded minus initial inoculum, (B) intracellular surviving minus adhered and invaded, and (C) intracellular growth minus survival upon exposure to an established population of *L. reuteri* ATCC PTA 6475 producing reuterin (PTA 6475+40 mM gly) or unable to produce reuterin (Δ*pduC* +40 mM gly and PTA 6475 no gly). Significant differences between the treatments are indicated with different letters (a, b or c; p<0.05).

The importance of glycerol reduction and reuterin production for *L. reuteri* to protect against χ3339 infection was tested in an approach 2 gentamicin exclusion assay (Fig. 1). An overnight culture of wild-type *L. reuteri* ATCC PTA 6475 was suspended in fresh GTSF-2 medium supplemented with 40 mM glycerol and incubated in the presence of 3-D HT-29 cells to allow for *in situ* reuterin production. The average reuterin production was 2.8±1.0 mM (after 30 min) and 3.8±1.3 mM (after 1 h30). Negative controls were provided by incubating (1) a *pduC* knockout of ATCC PTA 6475 in GTSF-2 with 40 mM glycerol and (2) ATCC PTA 6475 in GTSF-2 without glycerol. Reuterin production by both negative controls was always below detection limit (0.8 mM for 3-HPA). Fig. 5A shows that the presence of both wild type and *pduC* knockout strains of *L. reuteri* ATCC PTA 6475 significantly reduced χ3339 adhesion and invasion, but that this reduction was significantly higher when reuterin was produced *in situ*. Interestingly, the presence of all *L. reuteri* strains during the first 1 h30 of the assay further reduced the relative survival of the invaded χ3339 population after 4 h (Fig. 5B), but stimulated χ3339 intracellular growth after 24 h (Fig. 5C) when compared to the *Salmonella* control. For these time points, no significant differences were observed between reuterin-producing *L. reuteri* populations and the negative *L. reuteri* controls.

### 3-D HT-29 Aggregate Morphology and Cell Viability

**Figure 6:**
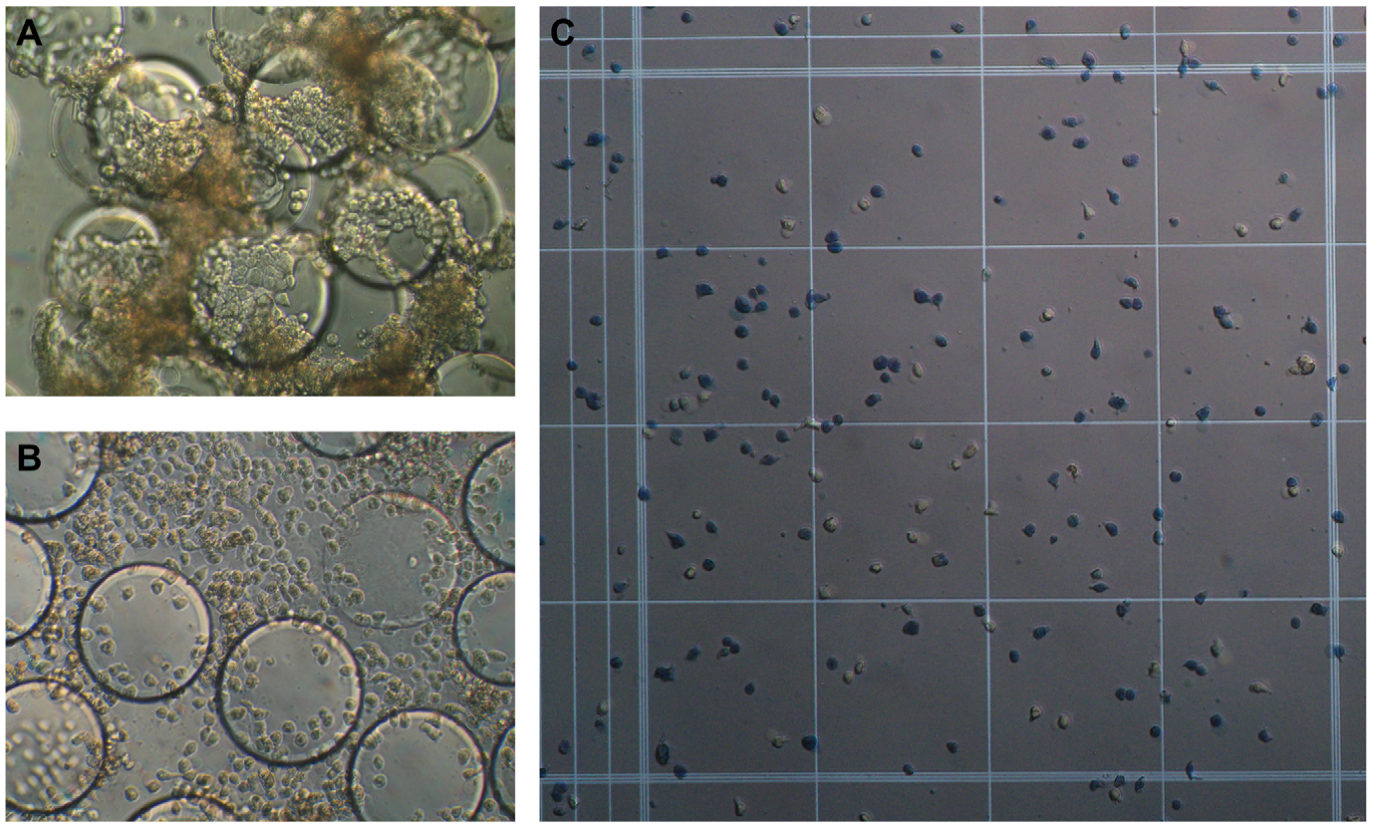
Long-term (24 h) exposure of 3-D HT-29 aggregates to 3-HPA (2.5–3 mM) results in loss of cell-cell contact and cell viability. (A) Aggregates of HT-29 colon cancer cells grown in three dimensions on porous collagen-coated microcarrier beads. The picture represents the situation for all treatments after 0, 1 and 4 h and the treatments with the *L. reuteri* supernatants without reuterin (SN- 1∶10) or containing low concentrations of reuterin (SN+1∶100 with 0.25 mM 3-HPA and SN+1∶1000 with 0.025 mM 3-HPA) after 24 h. (B) Example of a single cell suspension and naked porous collagen-coated microcarrier beads after 24 h of exposure to 3 mM 3-HPA or the 10-fold diluted reuterin-containing *L. reuteri* supernatant (SN+1∶10, 2.5 mM 3-HPA). (C) Example of live/dead counts of trypan blue treated single cell suspensions depicted in B with a hemocytometer. Dead cells are colored blue.

For every time point of the infection assays, separate wells were used to assess morphology of the 3-D HT-29 aggregates under a light microscope and to quantify cell viability after trypsinization and trypan blue staining. For both approaches 1 and 2 infection assays, a maximum of 10% dead cells was found for the aggregates exposed to all conditions after 1 and 4 h, both in the presence and absence of *S.* Typhimurium. An example for the morphology of these healthy aggregates is depicted in Fig. 6A. However, after 24 h a remarkable difference was observed in the morphology of aggregates exposed to 3 mM 3-HPA and the reuterin-containing *L. reuteri* supernatant (SN+1∶10, 2.5±0.4 mM 3-HPA), when compared to the other treatments and controls. Fig. 6B depicts the complete destruction of the 3-D structure of the aggregates into that of a single cell suspension. Moreover, 55±20% of these single cells were dead, as indicated by their absorption of trypan blue (Fig. 6C). Exposure to all other treatments of both approaches 1 and 2, and the untreated *S.* Typhimurium controls did not lead to any significant effect on cell viability at the 24 h exposure time point and resulted in 3-D morphology comparable to that shown in Fig. 6A.

## Discussion

Due to the increasing incidence of multidrug-resistant and highly invasive *Salmonella* strains, the development of new therapeutic treatments against human enteric salmonellosis becomes critical. In this study, we demonstrated the capacity of the reuterin-containing glycerol ferment of the probiotic and commensal species *L. reuteri* to significantly decrease adhesion and invasion, and intracellular survival of *S.* Typhimurium in a 3-D organotypic model of colonic epithelium. Moreover, it was found that an established *L. reuteri* population had an additional protective effect against χ3339 adhesion and invasion when it was able to convert glycerol to reuterin *in situ*. In contrast to conventional 2-D monolayers, our 3-D colon model was previously shown to respond to *S.* Typhimurium infection in key ways that reflect the infection process *in vivo*, including adherence, invasion, structural damage, cytokine production, and the lack of dependence on the SPI-1, SPI-2 and flagellar T3SS for *Salmonella* invasion [29], [30]. These findings highlight the relevance of our organotypic models for use as novel platforms to provide unique insight for development and screening of new therapies against *Salmonella* infection.

In approach 1 infection experiments, we compared the potential of the ferment of *L. reuteri* without and with glycerol to decrease colonization of the 3-D model colon epithelium by an actively growing χ3339 population (Fig. 3). Only the glycerol-supplemented ferment that contained reuterin at concentrations of 2.5±0.4 mM 3-HPA was found to significantly decrease colonization by χ3339. Higher dilutions of the reuterin-containing glycerol ferment (containing 0.25±0.04 mM 3-HPA and 0.025±0.004 mM 3-HPA) did not change the χ3339 infection process. However, the ferment without glycerol actually stimulated χ3339 intracellular survival and growth significantly. In this respect, the low concentrations of 3-HPA in the diluted reuterin-containing ferments (0.25±0.04 mM and 0.025±0.004 mM) could have counteracted the stimulatory effect on χ3339 intracellular survival and growth, as was observed for the ferment without glycerol or reuterin. This stimulatory effect might have been caused by the presence of low concentrations of acetic acid. This bacterial product was previously shown to stimulate the expression of SPI-1 T3SS genes at pH 6.7 but not at pH 8.0 [37], [38]. Since, the pH in our study was approximately 7.5, other unknown mechanisms were possibly involved in this stimulatory effect. Overall, these data illustrate the importance of glycerol and its conversion to reuterin for the inhibitory effects of the *L. reuteri* ferment towards χ3339 colonization.

Interestingly, the effects of pure 3-HPA and the *L. reuteri* ferment (both with and without glycerol) on the χ3339 population infecting host cells were found to be more profound than those in the absence of host cells (Fig. 4). Moreover, in the presence of the 3-D HT-29 cultures, pure 3-HPA exhibited a reduced ability to affect adherence and invasion, and intracellular survival of χ3339 populations as compared to the *L. reuteri* supernatant containing a similar amount of 3-HPA (2.5±0.4 mM 3-HPA). Interestingly, in the absence of host cells, both treatments resulted in an equal decrease of the χ3339 population. This suggests that the (combined) effect of *L. reuteri* metabolites on the ability of *S. *Typhimurium to colonize our well-differentiated models of intestinal epithelium may be enhanced due to (1) an increased sensitivity of χ3339 to these compounds in the context of an epithelial infection and/or (2) a host response to the bacterial metabolites of *L. reuteri* that affects the χ3339 infection process. The first mechanism could involve a direct or indirect effect of reuterin (alone or together with other metabolites) on *Salmonella* genes or their products that mediate colonization of the intestinal epithelium. While extremely limited information is available regarding the effect of reuterin on the mechanisms of *Salmonella* enteric pathogenesis, Kim *et al.* [39] found that a low concentration of reuterin (0.26 mM) is able to effectuate a moderate and short-term down-regulation of the SPI-1 T3SS genes *invA* and *hilA* without affecting the survival or growth of the *S.* Typhimurium culture after 8 h of exposure. The SPI-1 T3SS has been shown to be essential for *Salmonella* invasion into a variety of model host systems [33], [40]–[46]. However, the choice of host species alters *Salmonella* infection strategies, and it was previously shown that the SPI-1 T3SS is not required for *S.* Typhimurium invasion into our highly differentiated 3-D HT-29 colon models [29], [30], which is in agreement with studies reporting that SPI-1 is not required to cause enteropathogenesis *in vivo* [47]–[52]. Given the growing interest in the use of probiotics to maintain intestinal homeostasis and their potential to protect against enteric infection, identification of the mechanism(s) by which reuterin impacts intestinal colonization by *Salmonella* will likely become an area of expanding investigation. Central to the successful identification of these mechanisms will be the use of advanced intestinal models like those used in this study that exhibit key similarities to the responses of their *in vivo* parental tissues during a *Salmonella* infection.

The second mechanism would require the direct interaction of reuterin with the host cells, as 3-HPA was previously shown to bind to thiol groups and glutathione [11], [12]. In addition, reuterin can be spontaneously dehydrated to acrolein, which is a highly reactive toxin with a mutagenic potency comparable to that of formaldehyde [53], [54]. A possible reuterin interaction with host cells was furthermore supported in our study by the destruction of the 3-D aggregate structure and loss of cell viability within 24 h of exposure to both 3 mM pure 3-HPA and the reuterin-containing *L. reuteri* ferment (2.5±0.4 mM 3-HPA) (Fig. 6). This is the first study showing a reuterin effect on a model of intestinal epithelium. However, many *in vivo* trials support the safe administration of high doses of *L. reuteri* strains to full-term healthy infants younger than 4 months, healthy and even HIV-infected adults [55]–[58]. Given the reactive nature of reuterin with microbes and proteins, it could therefore be proposed that, *in vivo*, colonic reuterin interacts first with the luminal bacteria and bacteria colonizing the outer layer of the mucosal interface of the intestinal epithelium [59], [60], before it would affect the host. Further *in vitro* studies should carefully determine whether our observed reuterin effects are relevant in the presence of a complex gut microbiota and to what extent glycerol can be safely supplemented as a nutrient for the basal or enriched *L. reuteri* population in the human gut.

To further validate the safety and importance of the glycerol metabolism for *L. reuteri* to protect against intestinal *S.* Typhimurium infection, a second series of experiments was performed in which the model colon epithelium was infected with χ3339 in the presence of an established *L. reuteri* population that was either unable or stimulated to produce reuterin from glycerol (Fig. 5). In either case, *L. reuteri* was able to significantly reduce *S. *Typhimurium adhesion and invasion. This result was expected, as several research groups have demonstrated the capacity of *L. reuteri* to protect intestinal epithelial cells against inflammation and enteric infection by regulating the expression of pro- and anti-inflammatory cytokines [23]–[27] or to decrease *S.* Typhimurium adherence to Caco-2 cells [28]. However, we found that *L. reuteri* populations that were stimulated to convert glycerol to reuterin caused a significantly higher reduction of χ3339 adhesion and invasion as compared to populations that were not able to produce reuterin. Hence, in addition to *L. reuteri*’s protective effects via immunoregulatory modulation or competition for binding places [23]–[28], our results indicate that protection against *S.* Typhimurium could also occur via its capacity to produce reuterin from glycerol. This process was recently found to be important for the success and ecological behavior of *L. reuteri* strains isolated from the human gastrointestinal tract (GIT), poultry and to a lesser extent from pigs [21]. Moreover, glycerol is a potential nutrient source for *Salmonella* [61] and the latter was suggested to have acquired part of the genomic island for reuterin production via horizontal gene transfer, indicating that *L. reuteri* and *Salmonella* are potential competitors for the glycerol niche [20], [62]. Increasingly, metabolic pathways of the host and its associated microbiome are indicated to support growth of *S.* Typhimurium [63]. However, in this study we found an inhibitory microbial pathway that requires the presence of glycerol and *L. reuteri*. Previous studies indicated that the production of propionate and butyrate by the colon microbiota, but not acetate, reduces *Salmonella* invasion in the large intestine of mice [37], [38]. Together, these data suggest that management of the gut microbial metabolism towards such inhibitory pathways may be a new tool to protect against or treat *Salmonella* infections [38].

In conclusion, our results suggest that glycerol conversion to reuterin by *L. reuteri* might be an effective therapeutic approach to consider for protection against and treatment of intestinal *Salmonella* infections. However, prior to investigating its use as a potential antimicrobial agent in humans, both *in vitro* colonic fermentation studies [64] and *in vivo* model studies with reuterin would need to be performed to determine the effects and mechanisms of this compound on gastrointestinal health.
